# How Does ACR BI-RADS^®^ v2025 Change the Radiologist’s Approach? A Practical Guide Across Mammography, Ultrasound, and MRI: A Narrative Review

**DOI:** 10.3390/diagnostics16132135

**Published:** 2026-07-07

**Authors:** Ela Kaplan, Ahmet Burak Aydemir

**Affiliations:** Department of Radiology, Faculty of Medicine, Adiyaman University, Adiyaman 02040, Turkey; dr.ahmetburak06@gmail.com

**Keywords:** breast imaging reporting and data system, mammography, terminology as topic, ultrasonography, mammary, magnetic resonance imaging, contrast-enhanced mammography

## Abstract

Twelve years after the 2013 fifth edition, the American College of Radiology has released BI-RADS^®^ v2025, with substantial revisions across mammography, ultrasonography, MRI, and the newly independent contrast-enhanced mammography (CEM) section. This review compares the two editions and reads the main changes against the available evidence on diagnostic performance and reader agreement, rather than only cataloguing them. Examples featuring digital breast tomosynthesis, synthetic mammography, and automated whole-breast ultrasonography now appear throughout the modality sections. “Lobulated” has been added as a shape descriptor across all modalities, while “microlobulated” was dropped from the mammography margin list. Calcification terms shifted from etiology to morphology: “milk of calcium” became “layering,” “punctate” was folded into “round,” and “dystrophic” moved under “coarse.” Ultrasonography gained two new entries, “non-mass lesion” and “echogenic rind,” and on MRI, “initial phase” was renamed “early phase.” Category 6 was rewritten; therefore, surgical excision is recognized as one of several definitive local treatments, and an “uncoupled” principle now separates assessment from management. The auditing section folds Category 3 follow-up into basic auditing and adds a Method of Detection data field. Most supporting data, however, predate v2025, and reader agreement remains lower for newer descriptors such as non-mass enhancement; whether the revisions measurably improve reproducibility is still unproven. Integrating every high-sensitivity tool also brings more false positives, overdiagnosis, and cost; artificial intelligence and radiomics may help close the reproducibility gap, but resource-stratified, equitable implementation will be essential. With v2025, BI-RADS becomes a multimodality framework rather than a reporting lexicon alone.

## 1. Introduction

By the late 1980s, mammography reporting varied across institutions, with vague phrasing being common [1]. ACR accreditation began in 1986, the BI-RADS Committee was formed in 1988, and the first document appeared in 1992—built on three pillars: a literature-grounded lexicon, a standard reporting structure, and an audit framework [1].

Revisions followed in 1995, 1998, and 2003. The fourth edition replaced density with asymmetry, redefined microcalcification subgroups, split Category 4 into 4A/B/C, and extended the system to ultrasonography and MRI, with the MRI lexicon (morphologic and kinetic descriptors) entering with the 2003 edition [1]. MQSA mandated accreditation in 1992, and the 1999 regulation required a final BI-RADS category in every mammography report [1]; this has been Dutch law since 2000 [2].

The 2013 fifth edition was a systematic overhaul. Visual density estimates disagreed with automated volumetric calculations; therefore, percentage-based classification was replaced by four letter categories (a–d); vague terms like globular and tubular were dropped, and cross-modality alignment was reinforced [3]. Later use revealed limits: inter-observer agreement for non-mass enhancement (NME) on MRI was only moderate, and although clustered ring enhancement and segmental distribution carried predictive weight for malignancy, several descriptors remained subjective [4].

After twelve years, ACR BI-RADS^®^ v2025 brought structural changes. The rename from “Atlas” to “Manual” marks a shift in scope: the document now provides definitions, guidance, and a reporting framework rather than a visual lexicon alone, and versioning moved to publication year [5]. CEM moved from supplement to independent fifth section [5], consistent with evidence that it outperforms digital mammography and ultrasonography and matches MRI in select settings [6]. Lexicon terms are now ordered from least to most suspicious; reporting was harmonized across modalities; and a structured exam-indication system was added [7]. “Microlobulated” was dropped from mammography margins due to overlap with the “lobulated” shape, and DBT, SM, and AWBUS examples were embedded throughout [5].

These updates extend BI-RADS toward a clinical decision-support tool. As risk-stratified screening is redesigned for women with dense breasts and elevated risk, cross-modality consistency supports outcome tracking and decision-making, not just classification [8]. Early reproducibility data for the new CEM lexicon show particularly low inter-observer agreement for non-mass enhancement [9]. To date, most of the available literature addresses a single modality or concentrates on the structural updates, while the evidence behind the individual changes and their practical effect on reporting has received comparatively less attention. For radiologists adopting v2025, it therefore seems useful to consider not only what has changed but also how far the current evidence supports these revisions.

This review compares the 2013 fifth edition with v2025 across mammography, ultrasonography, magnetic resonance imaging, and the newly added contrast-enhanced mammography section (Table 1). Table 1 sets out the changes that affect reporting and cross-modality consistency; purely organizational differences are noted only briefly. Where possible, we relate the main changes to the available data on diagnostic performance and reader agreement and note their likely implications for routine reporting, using the Manual as a reference framework rather than as the sole source.

## 2. Methods

Two primary documents anchor this review: the 2013 ACR BI-RADS^®^ Atlas (5th Edition) and the ACR BI-RADS^®^ v2025 Manual. We searched PubMed for additional peer-reviewed work and hand-searched the reference lists of relevant articles published between 1998 and 2025. The PubMed search was last updated in 2025, and the full search strings are provided as Supplementary Materials. The search combined the terms “BI-RADS,” “Breast Imaging Reporting and Data System,” “breast lexicon,” “mammography,” “breast ultrasonography,” “breast MRI,” and “contrast-enhanced mammography,” in varying combinations. Articles were included on the basis of clinical relevance to multimodality breast-imaging terminology rather than under a formal systematic protocol. Inclusion was limited to peer-reviewed articles published in English, comprising original research, systematic reviews, meta-analyses, and consensus or society guidelines; conference abstracts, editorials, and isolated case reports were excluded unless they directly informed lexicon use. When a study is cited to support a statement about diagnostic performance or reader agreement, we note its design, sample size, and main limitations rather than reporting the figures on their own. The work is presented as a narrative synthesis; PRISMA 2020 reporting guidance does not apply. As this is a narrative review, we followed the SANRA (Scale for the Assessment of Narrative Review Articles) criteria, and the completed checklist is included as Supplementary Materials. During the preparation of this manuscript, the authors did not use any generative AI tool to write or generate the scientific text, and all data were produced by the authors. A generative AI tool (Claude Opus 4.8, Anthropic, San Francisco, CA, USA) was used only to format and arrange the layout of the Supplementary Materials File and its tables (Tables S1 and S2); it was not used to create the tables or generate any content. All data and content were created and verified by the authors, who take full responsibility for the work.

## 3. General Structural Changes

Each BI-RADS edition has revised lexicon content and document structure. Many of the differences between the 2013 fifth edition and v2025 are organizational, concerning format, length, and nomenclature, and carry little clinical weight; we set these aside and focus on the changes that bear on reporting and cross-modality consistency (Table 1).

The more consequential change is qualitative. DBT, synthetic mammography (SM), and AWBUS examples now appear across the modality sections, whereas the 2013 edition illustrated only standard mammography, conventional ultrasound, and MRI [5,10]. By placing these techniques inside the lexicon rather than in separate guidance, v2025 sets a common reference for describing and comparing findings obtained on them.

The 2013 atlas did not specify how lexicon terms should be ordered [10]. v2025 fills that gap by sequencing them, where applicable, from least to most suspicious [5], matching the risk-stratification logic of radiology practice. The change is presentational, with a plausible but as yet untested benefit for reporting consistency.

In 2013, the standard breast imaging report had seven components: study purpose and indication, breast composition, finding description, comparison with priors, final assessment, management recommendations, and unexpected findings [10]. v2025 keeps this framework, extends alignment across modalities, and adds the Structured Exam Indication system, built around three categories (Asymptomatic Screening, Diagnostic Workup, Active Breast Cancer) with defined subgroups and required fields [5]. Reporting therefore moves from modality- to indication-centered, providing the consistency needed for outcome tracking and auditing—a view echoed by the European Society of Radiology, which frames structured reporting as a value-driven quality tool [7].

Of these structural changes, the suspicion-level ordering and the structured indication system are the ones likely to reach day-to-day reporting and auditing; the rest are largely presentational. For institutions, the most important practical step is updating templates and information systems so the change reaches daily reporting.

## 4. Mammography

### 4.1. Breast Density (Composition)

Breast density classification has been revised in nearly every BI-RADS edition. The 2003 fourth edition used four numeric categories based on fibroglandular tissue percentage: <25% (Category 1, almost entirely fatty), 25–50% (Category 2, scattered fibroglandular densities), 50–75% (Category 3, heterogeneously dense), and >75% (Category 4, extremely dense) [10].

The 2013 fifth edition abandoned this scheme. Visual estimates disagreed with automated volume-calculation software, with the discrepancy widening as fibroglandular volume increased [3,11]; confusion between percent values and BI-RADS assessment categories also drove the change [3]. The new four-letter classification was (a) almost entirely fatty, (b) scattered fibroglandular density, (c) heterogeneously dense (may obscure small masses), and (d) extremely dense (lowers mammographic sensitivity) [10]. Under this masking-oriented logic, dense tissue concentrated in the upper portion that may obscure small masses can place a breast in Category C even when fibroglandular volume is below 50% [2].

v2025 preserved the basic classification with several additions. FDA mandatory patient notification, effective across all 50 U.S. states on 10 September 2024, was incorporated into the breast density section [12]. The FDA July 2025 alternative standard permits singular wording in unilateral mammography reports [5]. Categories C and D constitute dense breasts; the odds ratio for cancer in extremely dense breasts is 1.6, and DBT increases cancer detection in dense breasts from 4.5 to 5.8 per 1000 examinations [5]. Density reporting now functions as both a radiologic descriptor and a patient-centered communication tool with regulatory weight. Because the four-category scheme itself was largely carried over, the practical effect of the v2025 density section lies less in new terminology than in these communication requirements and their influence on supplemental-screening decisions. EUSOBI recommends MRI as the preferred supplemental screening modality in women with dense breasts and high risk, with ultrasonography as an alternative when MRI is unavailable [8]. The v2025 density-based notification system therefore extends beyond a mammography update and supports risk-stratified screening (Table 2).

### 4.2. Masses: Shape, Margin, Density

A mammographic mass is a three-dimensional, space-occupying lesion seen on two different projections [10]. In 2013, shape descriptors were narrowed to three: oval, round, irregular. The fourth-edition term “lobular” was removed because it risked confusion with the suspicious margin descriptor “microlobulated”; masses with two or three lobulations were folded into oval [3,10]. Margin assessment used five options—circumscribed, obscured, microlobulated, indistinct, spiculated—with microlobulated treated as suspicious [2]. A finding seen on only one projection was termed an asymmetry until a three dimensional character was confirmed [10].

v2025 reintroduced “lobulated” into the shape classification: an oval contour with one or more indentations is now described as lobulated and considered slightly more suspicious than oval [5]. This ranking is in keeping with earlier positive-predictive-value data, in which lobulated masses proved malignant somewhat more often than oval ones. The “microlobulated” margin term was removed because of overlap with the “lobulated” shape descriptor [5]. Lobulated-contour lesions therefore move from the margin to the shape category, which can influence the assigned suspicion level. Inter-observer agreement was only moderate in morphologically complex categories under the fifth-edition lexicon [4]; removing a difficult descriptor like “microlobulated” should improve standardization in clinical practice. With DBT, the features needed to define a lesion can be captured on a single projection, and reduced fibroglandular superposition makes “obscured” margin assessment easier [5]. The density classification (high, equal, low, fat-containing) was unchanged in both editions (Table 2).

### 4.3. Calcifications

Calcification classification has been revised repeatedly. The 2003 fourth edition placed calcifications into three groups: typically benign, intermediate concern, and high probability of malignancy [10]. In 2013, because both suspicious groups required biopsy, the two were merged under “suspicious morphology”; in the same revision, “eggshell” and “lucent-centered” were consolidated under “rim” [3].

In 2013, typically benign calcifications included skin, vascular, coarse/popcorn-like, large rod-like, round and punctate (<1 mm), rim, dystrophic, milk of calcium, and suture [10]. The suspicious morphology category contained amorphous (4B), coarse heterogeneous (4B), fine pleomorphic (4B), and fine linear/linear-branching (4C). Multiple studies have confirmed that fine linear/branching morphology carries a substantially higher malignancy risk than other suspicious descriptors [13,14]. Reported positive predictive values are on the order of 50–70% for fine linear or linear-branching calcifications, against roughly 7–29% for amorphous, coarse heterogeneous, and fine pleomorphic morphology [13,14]. The upper size limit for clustered distribution was raised in 2013 from 1 cm to 2 cm [2].

**Figure 1:**
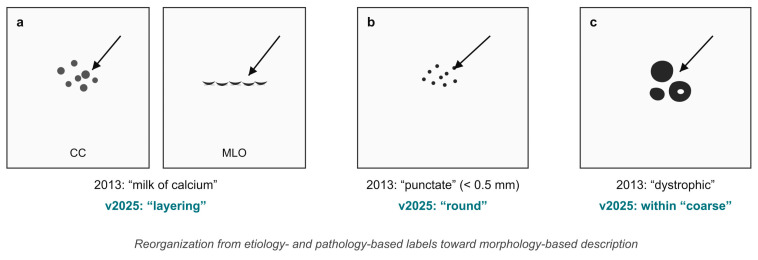
Calcification terminology in the 2013 and v2025 BI-RADS lexicons for three morphologies: (**a**) “milk of calcium” designated “layering,” shown on paired cranio-caudal and medio-lateral oblique projections, where the dependent, layering appearance is evident on the upright (MLO) view; (**b**) “punctate” folded into “round”; and (**c**) “dystrophic” assessed within the “coarse” category. The panels illustrate the reorganization from etiology- and pathology-based labels toward morphology-based description. Arrows indicate the calcifications. CC, cranio-caudal; MLO, medio-lateral oblique.

v2025 introduced three terminology revisions. The crescent-shaped MLO appearance and indistinct CC appearance led to “milk of calcium” being renamed “layering” [5]. Measuring calcifications below 0.5 mm is impractical and does not affect clinical decisions; therefore, “punctate” was folded into “round” [5]. “Dystrophic” was placed under “coarse,” now described by shape and size within that category [5]. The distribution classification (diffuse, regional, grouped, linear, segmental) was largely preserved. Calcification terminology has therefore moved from etiology- and pathology-based labels to morphology-based description (Figure 1), which should reduce subjective interpretation and support consistent terminology across modalities (Table 2). Since the suspicious categories that prompt biopsy were left unchanged, these revisions are largely terminological and are unlikely to alter management, although they may make the labeling of benign calcifications more consistent.

### 4.4. Asymmetries

Unilateral tissue accumulations that do not meet mass criteria are termed asymmetries. The 2013 edition defined four types. Asymmetry is unmatched tissue on only one projection, usually normal tissue overlap. Focal asymmetry—seen on both projections, without convex contour or central density and with interspersed fat—has a positive predictive value of 7.4–12.8% in screening and 19.7–26.7% in diagnostic settings [2,5]. Global asymmetry covers ≥1 quadrant and lacks a corresponding contralateral structure; usually a normal variant, it should be assessed alongside clinical findings. Developing asymmetry was a focal asymmetry that became newly apparent, larger, or more conspicuous than on prior studies, and was treated as suspicious and potentially requiring biopsy [10]. Mammographic detection rate is approximately 1.6 per 1000, with biopsy malignancy rates up to 42.9% [15].

v2025 removed “developing asymmetry” as an independent descriptor. The Manual states that this does not reduce its clinical importance: new or more conspicuous focal asymmetry should still be evaluated as suspicious and may warrant biopsy [5]. The change is terminological rather than conceptual; new or increasing focal asymmetry remains an indication for biopsy. Asymmetries have concave borders with interspersed fat, while masses have convex borders and central density greater than the periphery—a distinction valid in both editions [2,10] (Table 2).

### 4.5. Architectural Distortion, Skin Lesions, Dilated Duct, and Special Cases

In 2013, the mammography section gave independent categories to findings previously grouped under “special cases” or “associated features,” including intramammary lymph node, skin lesion, and solitary dilated duct. Solitary dilated duct earned its own category because malignancy rates in isolated cases without accompanying suspicious findings reach 9.5% in large-scale studies [16]. Architectural distortion was defined as disruption of normal breast architecture without an evident mass; thin lines radiating from a point or focal retraction at parenchymal edges raise the differential between scar and carcinoma [10].

v2025 retained the basic definitions but revised their clinical context. The Manual emphasizes that DBT detects architectural distortion far more often than conventional two-dimensional mammography; principal associated pathologies include radial scar, invasive lobular and ductal carcinoma, and sclerosing adenosis [5]. Imaging protocols after neoadjuvant therapy and supplementary screening for high-risk individuals were expanded. The recommendation to set institutional policy on radiopaque markers appears in both editions [5,10]. These updates do not change the clinical meaning of architectural distortion but increase detection frequency, with implications for follow-up and workload; with DBT now widespread, the threshold for including architectural distortion in the differential has decreased substantially.

The mammography-section changes fall into three groupings. Calcification revisions illustrate the move from pathology- and etiology-based to morphology-based terminology: milk of calcium renamed layering, punctate folded into round, dystrophic placed under coarse. The second is cross-modality shape alignment, with “lobulated” added and “microlobulated” removed to resolve overlap. The third is the integration of legal and patient-centered communication, reinforced by FDA notification requirements and risk-stratified supplementary screening guidance now embedded in the Manual. These revisions should reduce inter-observer variation, improve report consistency, and standardize patient communication.

## 5. Ultrasonography

### 5.1. Mass Shape: “Lobulated” Added Across All Modalities

In 2013, ultrasound mass descriptors were arranged largely to align with the mammography lexicon. Shape options were limited to three—oval, round, irregular—and “lobulated” was absent in both modalities [10]. Margin assessment used a circumscribed/not-circumscribed distinction, with not-circumscribed subgroups defined as indistinct, angular, microlobulated, and spiculated [10]. The orientation parameter, introduced for the first time in 2013, had two options: parallel to skin (benign-leaning) and not parallel to skin (formerly “taller-than-wide,” considered suspicious) [2].

v2025 added “lobulated” to shape classification—an oval contour with one or more indentations [5]. Placing it alongside oval, round, and irregular allows for a more precise description and creates a shared shape vocabulary across mammography, ultrasonography, and MRI [5]. Radiologists assessing the same lesion through different modalities can therefore communicate in common terms, supporting consistency in multidisciplinary tumor boards (Table 3).

### 5.2. A Brand-New Category: Non-Mass Lesion

In 2013, the ultrasound lexicon was built around mass and cyst descriptions, with no independent category for focal lesions that did not meet mass criteria but could be distinguished from normal breast tissue [10].

v2025 introduces “non-mass lesion” as a new finding category for focal lesions that do not satisfy mass criteria but are distinguishable from heterogeneous breast tissue [5]. The category is the ultrasound counterpart of focal asymmetry on mammography and non-mass enhancement on MRI, particularly valuable in non-mass-forming tumors such as invasive lobular carcinoma and in situ cancers; one practical aim is supporting ultrasound-guided biopsy of findings detected on DBT, MBI, and CEM [5]. Distribution (focal, linear, segmental, regional, diffuse) and echo pattern (anechoic, hypoechoic, heterogeneous, hyperechoic) define the descriptor. On MRI, inter-observer agreement for non-mass enhancement can remain moderate (κ 0.67–0.69) within distribution and internal enhancement subcategories [4], and similar challenges are likely on ultrasound; systematic training and standardized reporting templates should help as the category comes into wider use (Table 3).

### 5.3. Echo Pattern: “Mixed Solid and Cystic” Reinstated

In 2013, mass echo pattern was defined with six options: anechoic, hypoechoic, isoechoic, hyperechoic, complex cystic and solid, and heterogeneous; “mixed solid and cystic” was absent as an independent descriptor [10].

v2025 reinstated “mixed solid and cystic” to emphasize the relative importance of the solid component [5]. The distinction was clarified: “mixed solid and cystic” is used for lesions clearly containing both components, while “complex cystic and solid” remains specific to thick-walled cysts, septated cysts, or cysts containing solid nodules [5]. The two descriptors direct clinical attention to any solid component when present (Table 3).

### 5.4. Associated Features: “Echogenic Rind” Added

In 2013, ultrasound associated features comprised architectural distortion, ductal changes, skin thickening, skin retraction, edema, vascularity (absent/internal/rim), and elastography [10]. Elastography was new in this edition, with tissue stiffness rated soft, intermediate, or hard. Compression elastography combined with B-mode ultrasound offered high sensitivity and specificity [17] (sensitivity 99.0% and specificity 91.5% in a community-based series), and shear-wave elastography is more reproducible than strain elastography [18] and, when the two were compared directly, separated benign from malignant lesions more accurately than B-mode alone (AUC 0.928 versus 0.851) [18].

v2025 added “echogenic rind” to the associated features list—an echogenic halo around a lesion that captures the surrounding tissue reaction seen in some invasive cancers [5]. Elastography terminology was unchanged. The radiologist can now describe a lesion’s relationship with adjacent tissue using a defined term, which should improve differential evaluation, particularly for invasive cancers (Table 3).

### 5.5. Lymph Node Assessment: Detailed Expansion

In 2013, a normal intramammary lymph node was described as a structure with fatty hilum, oval or kidney shape, and generally <1 cm in size. Axillary lymph nodes were addressed under “special cases,” and morphologic criteria for tumor involvement were only briefly reported [10].

v2025 expanded lymph node assessment substantially [5]. Axillary levels I–II–III, internal mammary, supraclavicular, and infraclavicular basins are now presented as the regional nodal basins, each linked to its role in TNM nodal staging. Normal node criteria specify cortical thickness ≤3 mm and a clear fatty hilum. Suspicious morphologic changes—cortical thickening, eccentric cortex, hilar effacement, and rounding—were also defined [5]. Linking basins to TNM staging gives multidisciplinary teams standardized ultrasound data directly relevant to clinical decision-making (Table 3).

Four threads run through the ultrasound section. Cross-modality alignment was strengthened with “lobulated” added to the US lexicon. The non-mass lesion category extends ultrasound beyond mass-based description. “Mixed solid and cystic” and “echogenic rind” added diagnostic detail. Lymph node assessment was tied to TNM staging. The ultrasound report now supports lesion description, cross-modality communication, staging, and clinical decision-making.

## 6. Magnetic Resonance Imaging (MRI)

### 6.1. Mass Shape: “Lobulated” Added to the MRI Lexicon

MRI mass descriptors have been aligned with mammography and ultrasonography in each edition. In 2013, MRI mass shape had three options: oval, round, irregular. The “lobular” term from the fourth edition was dropped as functionally equivalent to “oval” [3]. The circumscribed margin terminology was renamed from “smooth” to “circumscribed”; “irregular” and “spiculated” remained in the not-circumscribed group [3]. Internal enhancement included homogeneous, heterogeneous, rim enhancement, and dark internal septations; T2 signal intensity was not part of the mass description [10].

v2025 reintroduced “lobulated”—an oval contour with one or more indentations, considered slightly more suspicious than oval [5]. With lobulated alongside oval, round, and irregular, the three modalities now share a common shape vocabulary [5]. T2 signal intensity was added as a new mass parameter, with two options: hyperintense and not hyperintense [5]. The addition introduces a signal-based parameter to MRI lesion description, fitting the multiparametric character that sets MRI apart from the other two modalities (Table 4).

### 6.2. Kinetic Curve: “Initial Phase” → “Early Phase”

Kinetic curve assessment on dynamic contrast-enhanced MRI complements morphologic descriptors. In 2013, the early phase had three options (slow, medium, rapid) and was termed “initial phase” [10]. The late phase included persistent, plateau, and washout [3]. Rising BPE reduces DCE-MRI sensitivity for breast cancer detection; therefore, reporting the BPE level is mandatory [19]. In one series, DCE-MRI sensitivity fell from 99% in breasts with minimal or mild BPE to 88% in those with moderate or marked BPE, a difference of 11% (*p* = 0.0058) [19].

v2025 renamed “initial phase” to “early phase” [5]. With fast MRI protocols, multiple early time points after contrast injection can be acquired, leaving “initial” ambiguous [5]. Early-phase options remained slow, medium, and rapid, and late-phase terminology was unchanged [5]. The same edition also introduced abbreviated MRI protocols. The renaming is more than terminological—it addresses the ambiguity introduced by fast protocols. Defining an “early phase” interval supports consistent reporting while allowing for protocol variation; the introduction of abbreviated MRI in the same edition fits this push for protocol-flexible terminology (Table 4).

### 6.3. Non-Mass Enhancement (NME) Updates

NME describes non-mass enhancement without convex margins on MRI. In 2013, “clustered ring”—periductal stromal enhancement with diagnostic value—joined the internal enhancement pattern list [3]. The “ductal” distribution descriptor and the “reticular/dendritic” and “stippled/punctate” internal enhancement patterns were dropped due to low usage [3]. “Symmetric” and “asymmetric” remained within the NME category in 2013 [10].

v2025 moved “symmetric” and “asymmetric” to the background parenchymal enhancement (BPE) category; therefore, NME is now described by morphologic distribution and internal enhancement pattern [5]. The distribution classes (diffuse, regional, focal, linear, segmental) and internal enhancement options (homogeneous, heterogeneous, clumped, clustered ring) were retained [5]. NME thus serves as a morphologic descriptor and BPE as a background assessment, with the overlap removed. A prospective study using the fifth-edition lexicon found that clustered ring internal enhancement and segmental distribution contributed meaningfully to malignancy prediction (PPV 53.85% and 62.5%, reaching 66.67% in combination), with moderate inter-observer agreement (κ = 0.67–0.69) for both subgroups [4]. Simplifying the NME definition should reduce these difficulties and improve descriptor consistency (Table 4).

### 6.4. Implant Assessment

The 2013 fifth edition substantially expanded implant assessment terminology, which had been limited in earlier editions. Retroglandular and retropectoral defined implant location. “Focal bulge” was introduced for abnormal contour; intracapsular findings included radial folds, subcapsular line, keyhole sign, and linguine sign. Extracapsular silicone descriptors covered lymph node and breast localization, water droplets, and peri-implant fluid [3]. Lumen type (single, double, other) and material classification (saline, silicone with intact or ruptured states, and other) also entered the system [3].

v2025 kept the 2013 implant lexicon—visual examples were updated with current high-quality MRI images, and practical guidance for abbreviated implant MRI protocols was added [5]. Leaving the lexicon unchanged avoids disrupting terminology already settled in clinical practice; v2025 updated visuals and protocol guidance rather than the words themselves (Table 4).

Several threads run through the MRI section. Cross-modality shape alignment was strengthened by adding “lobulated.” T2 signal intensity introduces a functional parameter to lesion characterization. Renaming “initial phase” to “early phase” alongside abbreviated MRI gives the lexicon room for protocol variation. Moving “symmetric” and “asymmetric” out of NME removed overlap with BPE. The MRI lexicon now extends beyond pure morphology, with protocol flexibility, cross-modality alignment, and interpretive simplification, though systematic education and standardized reporting templates are still needed where inter-observer agreement is only moderate, as in NME.

## 7. Contrast-Enhanced Mammography (CEM): A New Independent Section

CEM acquires low-energy (LE) and recombined (RC) images after intravenous iodinated contrast injection, providing anatomic and physiologic information in a single examination. In the 2013 fifth edition, CEM was not part of the principal lexicon because clinical use was still limited; it appeared only as an atlas supplement [10].

v2025 includes CEM as an independent section (pp. 725–799) at the same structural level as mammography, ultrasonography, and MRI, turning the manual into a five-section reference [5]. The move from atlas supplement to independent section tracks the modality’s growing evidence base. Recent retrospective and prospective studies and meta-analyses have shown that CEM substantially improves diagnostic performance over digital mammography and ultrasonography and produces results comparable to breast MRI in select clinical settings [6]; therefore, its inclusion in v2025 matches a shift already underway in clinical practice.

CEM produces two image types. LE images match standard digital mammography in anatomic quality and use the mammography lexicon (mass, calcification, asymmetry) [5]. RC images capture contrast uptake and physiologic assessment, with their own dedicated lexicon [5]. The two-lexicon structure lets the CEM report combine mammographic and MRI-like elements in one examination.

On RC images, BPE is described at four levels (minimal, mild, moderate, marked), with symmetric and asymmetric options [5]. RC findings fall into three categories. Masses are described by shape (oval, lobulated, round, irregular), margin (circumscribed; not-circumscribed: indistinct or spiculated), and internal enhancement (homogeneous, heterogeneous, rim enhancement) [5]. NME distribution descriptors are diffuse, regional, focal, linear, and segmental, with “multiple regions” removed for MRI alignment [5]. Enhancing asymmetry—an enhancement area on a single projection—is the third finding category [5]. The lexicon adopts “lobulated” across modalities, and “indistinct” was preferred over “irregular” for RC margins to match mammography and MRI [5]. An early multi-reader study of the CEM lexicon reported moderate-to-substantial agreement for most features, including lesion type on low-energy images (κ 0.65) and the type of enhancement on recombined images (κ 0.66), as well as for the mass enhancement descriptors of shape, margin, and internal pattern (κ 0.52 to 0.62). Agreement was lower for the newer recombined descriptors, namely non-mass enhancement (κ 0.39 to 0.42) and enhancing asymmetry (κ 0.25), although the readers’ diagnostic performance was preserved [9]. The lexicon will likely need structured training materials and calibration exercises as it enters routine clinical use.

Three points capture the elevation of CEM to independent-section status. Growing real-world clinical use and the recent body of evidence are now formally recognized in the manual [6]. A CEM-specific lexicon was systematically defined for the first time, with cross-modality alignment maintained. Initial reproducibility data guide integration of the lexicon into clinical practice and identify areas needing improvement [9]. CEM is therefore an integral part of the BI-RADS cross-modality reporting structure rather than a newly added modality.

## 8. Assessment Categories (BI-RADS 0–6)

BI-RADS assessment categories link radiologic findings to management decisions across modalities. Each category covers a defined probability-of-malignancy range with a standard management recommendation, supporting radiologist–clinician communication and supplying data to auditing processes [10].

In 2013, category definitions were largely standardized. Category 0 marks an incomplete assessment requiring additional imaging or comparison with priors [10]. Category 1 is a negative assessment, and Category 2 records a benign finding; both recommend routine screening and carry essentially 0% malignancy probability [2,10]. Category 3 covers a probably benign finding (>0% to ≤2% probability), with six-month short-interval follow-up as primary management. It cannot be used in screening mammography and is assigned only after full diagnostic evaluation following BI-RADS 0 [2,10]. Early PPV studies found no malignancy in biopsied Category 3 lesions, supporting the basis of the category [20].

Category 4, the broadest in practice, denotes a suspicious abnormality covering the 2–95% probability range, split into three subgroups: 4A (low, >2% to ≤10%), 4B (moderate, >10% to ≤50%), 4C (high, >50% to <95%); tissue diagnosis is indicated in all [10]. PPV studies of microcalcification descriptors support this split, with fine linear/branching morphology carrying substantially higher malignancy risk than other suspicious descriptors [14]. The probability ranges are also borne out at the category level: in one large series the positive predictive value was about 34% for Category 4 and 81% for Category 5 [20]. Category 5 describes a finding highly suggestive of malignancy (≥95%), and Category 6 denotes biopsy-proven malignancy; in 2013, Category 6 management was “surgical excision when appropriate” [10].

In v2025, the definitions and probability ranges for Categories 0 through 5 were preserved [5]. Three updates stand out.

The first update concerns Category 6 management wording. With neoadjuvant systemic therapy, radiofrequency ablation, and other minimally invasive local therapies entering breast cancer management, the recommendation was rewritten as “clinical follow-up; definitive local therapy (usually surgical, but not the only option)” [5]. The new wording removes surgical excision as the sole definitive option, opening room for multidisciplinary team decisions and turning radiologist–clinician communication into a more flexible management discussion.

The second update codifies the “uncoupled” assessment-management principle: when multiple findings are present, each is evaluated separately, the final category is determined by the most suspicious finding, and assessment can be configured independently from the management recommendation [5]. Objective classification of imaging findings (assessment) is therefore separated from the decision shaped by clinical context (management). The two are not always linked; multidisciplinary opinion and individual patient factors can enter management selection directly; therefore, the same BI-RADS category can lead to different management recommendations across clinical contexts.

The third update links categories to structured clinical indications. Asymptomatic Screening, Diagnostic Workup, and Active Breast Cancer are the three primary indications; combining them with the assessment category structures outcome tracking and cross-modality comparison [5]. The indication-centered approach aligns with risk-stratified screening, where modality choice is driven by clinical context and individual risk [8]. Active Breast Cancer brings post-diagnosis follow-up, neoadjuvant therapy response assessment, and recurrence surveillance under a single heading, adding clinical context to reporting (Table 5).

## 9. Auditing and Outcomes Monitoring (AOM)

Auditing and outcomes monitoring is one of the BI-RADS founding principles, defining the methodology and metrics for measuring a breast imaging practice’s clinical performance and giving the system a quality-assurance role alongside its reporting role [10].

In the 2013 fifth edition, the section was titled “Follow-up and Outcomes Monitoring” [10]. Its principal metrics were cancer detection rate (CDR; cancers per 1000 examinations), abnormal interpretation rate (AIR), recall rate, three forms of positive predictive value (PPV_1_, PPV_2_, PPV_3_ for all positive screens, biopsy recommendations, and biopsied examinations, respectively), and sensitivity/specificity [10]. The framework supported tracking of category distributions, recall and cancer-detection performance, and benchmarking against national and international values.

v2025 renamed the section “Auditing and Outcomes Monitoring (AOM)” [5]. The change is more than terminological—“auditing” captures the active, performance-oriented character better than “follow-up” [5]. The section now extends beyond recording past events to a structured approach for continuous improvement of clinical practice.

v2025 added six elements to the AOM section. The first is the inclusion of Category 3 findings in basic auditing. The 2013 basic audit focused on Categories 4, 5, 6 with biopsy yield; v2025 brought follow-up outcomes for probably benign lesions into systematic quality assurance, making Category 3 use trackable [5]. Because Category 3 is frequently used but interpretively broad, auditing it allows for numerical tracking of overuse, unnecessary follow-up burden, and missed malignancy, and supports institutional evaluation of category-assignment behavior.

The second addition expands MRI auditing for disease-extent assessment, addressing the growing role of MRI in neoadjuvant treatment planning and staging; the “More Complete Audit” now covers additional findings detected on disease-extent breast MRI examinations [5].

The third and most notable addition is Method of Detection (MOD), introduced for the first time in v2025 to record the modality by which a cancer was first detected. Combined with structured clinical indications, MOD enables cross-modality outcome comparison and quantifies the contribution of supplemental screening relative to standard mammography at institutional and national levels [5]. It is the first structured, modality-neutral field for measuring how much US, MRI, CEM, and AWBUS add to standard mammography. Alongside risk-stratified screening recommendations that prefer MRI as supplemental screening in dense-breasted women and accept ultrasound when MRI is unavailable, MOD generates the data needed to assess which modality benefits which patient group [8].

The fourth addition is the “What Not to Audit” subsection, which clarifies which examination types should be excluded from audit calculations and addresses commonly misunderstood scenarios, supporting consistent and comparable audit data [5]. It is a standardization step aimed at reducing methodological errors.

The fifth and sixth additions are paired. Screening and diagnostic examination definitions, largely mammography-centered in 2013, were rewritten as modality-neutral; therefore, consistent classification applies to ultrasonography, MRI, CEM, and future modalities [5]. Benchmark values for cancer detection rates and PPV ranges across mammography, ultrasonography, and MRI were updated with current data [5]. Modality-neutral definitions keep the AOM framework applicable to future imaging technologies, in line with the European Society of Radiology’s value-driven vision for structured reporting [7].

Several threads run through the AOM updates. The “Auditing” rename signals a move from reactive monitoring to proactive performance measurement. Category 3, MRI disease-extent, and MOD widen the audit scope; the “What Not to Audit” subsection reinforces methodological consistency; and the modality-neutral framework prepares the system for future imaging. AOM now supports cross-modality comparative analysis, technology-effectiveness assessment, and risk-stratified screening strategies alongside its post-reporting role.

### Clinical Scenario: The Practical Reflection of v2025

**Figure 2:**
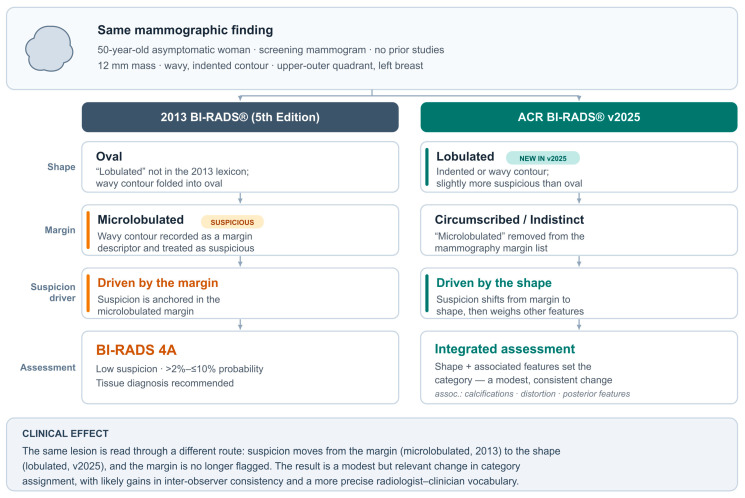
Side-by-side application of the 2013 (fifth-edition) and ACR BI-RADS^®^ v2025 lexicons to the same screening finding. A 50-year-old asymptomatic woman has a 12-mm mass with a wavy, indented contour in the upper-outer left breast and no prior studies. Under the 2013 lexicon, “lobulated” is unavailable; therefore, the shape is recorded as oval and the wavy contour is captured by the “microlobulated” margin descriptor; because microlobulated is a suspicious margin, the finding is assigned BI-RADS 4A and tissue diagnosis is recommended [10]. Under v2025, the shape is “lobulated”—slightly more suspicious than oval—and the margin becomes circumscribed or indistinct because “microlobulated” has been removed [5]. The driver of suspicion therefore moves from the margin to the shape, and the final category is reached by integrating the lobulated shape with associated features such as calcifications, architectural distortion, and posterior features rather than from the margin alone. The net effect is a modest but relevant change in category assignment, with likely gains in inter-observer consistency for lobulated-contour lesions and a more precise vocabulary for radiologist–clinician communication [4]. *BI-RADS, Breast Imaging Reporting and Data System.*

Consider a 50-year-old asymptomatic woman whose annual screening mammogram shows a 12-mm mass with a wavy, indented contour in the upper outer left breast, with no priors. This single finding can be followed through both lexicons in parallel (Figure 2). Under the 2013 lexicon, with “lobulated” absent, the shape falls under oval, and the wavy contour is captured with the “microlobulated” margin descriptor; because microlobulated is suspicious, the assessment is most likely BI-RADS 4A with tissue diagnosis [10]. Under v2025, the shape is “lobulated”—slightly more suspicious than oval—and the margin moves to circumscribed or indistinct because “microlobulated” was removed [5]. The suspicion level shifts from margin to shape, and the final category is reached through a broader reading that also weighs associated features such as calcifications, architectural distortion, and posterior features. The change in the driver of suspicion, from margin to shape, and its effect on the final category are set out side by side (Figure 2). The clinical effect is a modest but relevant change in category assignment, with likely gains in inter-observer consistency for lobulated-contour lesions and a more precise vocabulary in radiologist–clinician communication [4].

## 10. Discussion/Current Challenges and Future Perspectives

ACR BI-RADS^®^ v2025 is a substantial revision published after a twelve-year interval, notable for its scope and for the way the changes support each other.

The most visible change is the integration of new technologies. DBT, synthetic mammography, and AWBUS now appear as embedded clinical examples across modality sections rather than as separate notes. CEM moved from atlas supplement to independent fifth section [5], in step with its growing clinical role. CEM substantially improves diagnostic performance over digital mammography and ultrasonography and produces results comparable to breast MRI in select settings [6], while early reproducibility studies are already identifying where the CEM-specific lexicon needs improvement [9].

Terminology changes look modest but are clinically relevant. Adding “lobulated” to all three modalities while removing “microlobulated” from mammography is intended to reduce terminology differences between reports examining the same lesion with different methods [5]. Inter-observer agreement could remain moderate in morphologically complex categories under the fifth-edition lexicon [4], and the v2025 simplifications should improve report consistency in daily practice. It should be acknowledged, however, that most of this evidence predates v2025: the agreement and predictive-value figures cited throughout this review come from the fifth-edition lexicon or from the first CEM lexicon, not from v2025 itself; therefore, the expected gains in consistency remain reasonable inferences rather than demonstrated outcomes (Table 6). The reliance on older data is partly unavoidable: these PPVs predate tomosynthesis and have not been reproduced for the new lexicon [20]. A gap therefore remains for DBT, which v2025 embeds throughout while these figures come from two-dimensional mammography [5].

A few tensions in the underlying evidence are worth noting. The data supporting individual descriptor changes are uneven: for calcifications the malignancy gradient is well documented, with fine linear or linear-branching morphology reaching positive predictive values of roughly 50–70% against 7–29% for the other suspicious descriptors [13,14], whereas for newer entries such as “echogenic rind” and the ultrasound “non-mass lesion” category direct performance data are still scarce. Reproducibility is also uneven across modalities; on contrast-enhanced mammography, for example, reader agreement is moderate to substantial for mass descriptors but only fair for non-mass enhancement and enhancing asymmetry (κ 0.25–0.42), even though diagnostic performance was maintained [9]. These are the areas where v2025 is most likely to need further validation.

Among the reporting-structure changes, the structured exam-indication system, particularly the Active Breast Cancer category, will require the most adjustment in practice [5]. The removal of surgery-centered language from the Category 6 wording should be communicated directly to multidisciplinary teams. The “uncoupled” assessment-management principle is the more far-reaching of these changes, and it carries practical risks. By allowing one category to carry different management recommendations, it loosens a link clinicians have long relied on, and reports may be harder to interpret across institutions. Variation can now enter at the management level even when readers agree on the category, since management may be shaped by clinical context and multidisciplinary input [5]. It also has a medicolegal dimension: when a suspicious category is paired with a more conservative recommendation, the reasoning should be documented, since such a divergence is harder to defend than a recommendation that follows directly from the category.

On the auditing side, MOD will likely become an important data field for cross-modality comparative analyses, and including Category 3 findings in basic auditing is a structural gain that should improve reporting reliability over time [5]. MOD allows for a numerical assessment of which supplemental modality (MRI, US, or CEM) contributes most to standard mammography in women with dense breasts and elevated risk, giving institutions a way to track the effectiveness of risk-stratified screening recommendations [8].

Integration priorities will vary by institution; for most centers the starting points are updating CEM reporting templates to the v2025 lexicon and translating the microcalcification terminology changes into daily practice.

The move to electronic format frees future revisions from ten-year cycles [1]. Given the pace of breast imaging technology, this flexibility may prove one of the v2025 most valuable features. Looking ahead, the most useful next steps would be prospective studies that compare inter-observer agreement and diagnostic performance under the v2025 and fifth-edition lexicons directly, together with multicentre work on the descriptors that currently lack validation, such as echogenic rind, the ultrasound non-mass lesion category, and the recombined CEM descriptors.

### 10.1. Artificial Intelligence and Radiomics: Narrowing the Reproducibility Gap

A recurring theme across the modality sections of this review is that the descriptors most in need of standardization—non-mass enhancement on MRI and contrast-enhanced mammography, the new ultrasound non-mass lesion, and breast density—are also those for which human reading is least reproducible. Artificial intelligence (AI) is the most plausible external lever for this problem, and v2025 arrives at a point where the supporting evidence is becoming concrete. Breast density illustrates the limitation of visual assessment most clearly: even when weighted inter-reader agreement is nominally good (κw 0.80–0.84), discordance between two radiologists scoring the same examinations can reach 32.4%, which is enough to make density scoring by an individual reader unreliable for selecting women into supplemental or risk-adapted pathways [21]. Automated volumetric density software, such as Volpara and Quantra, was developed precisely to remove this subjectivity [21], and FDA-cleared AI applications have already entered routine workflows [22]. A representative example is the iCAD FDA-cleared ProFound Density [23]; more broadly, deep-learning models that assign BI-RADS density categories have been shown to improve consistency and reduce the inter-observer variability of visual scoring [24,25]. For the descriptor that this review repeatedly identifies as the weakest link, non-mass enhancement, a deep-learning model (ResNet50) applied to dynamic contrast-enhanced MRI distinguished benign from malignant lesions with an area under the curve of 0.91, statistically equivalent to a highly experienced radiologist (0.89) and clearly superior to a resident (0.64) [26]. Comparable signals are emerging for the contrast-based modalities that v2025 now emphasizes: machine- and deep-learning models built on radiomic features extracted from CEM and DCE-MRI separated benign from malignant lesions with an accuracy and area under the curve of approximately 0.91 [27]. Read together, these results suggest that AI and radiomics could supply the reproducibility that lexicon alone cannot guarantee, particularly for non-mass and contrast-enhancement findings, and could extend consistent interpretation to less experienced readers [26]. The qualification raised throughout this review applies here as well; however, such tools carry their own requirements for prospective validation, explainability, regulatory clearance, and equitable access [22], and they are best understood as a complement to the v2025 framework rather than a substitute for it.

### 10.2. The Cost of Higher Sensitivity: False Positives, Overdiagnosis, and Health Economics

The same breadth that makes v2025 attractive also carries a cost the Manual does not dwell on. Integrating every high-sensitivity tool—the new ultrasound non-mass lesion category and the now-independent CEM section—raises detection, but gains in sensitivity are routinely offset by reductions in specificity, with downstream false-positive recalls, benign biopsies, and resource strain [28]. The supplemental ultrasound literature is the clearest precedent. In the ACRIN 6666 trial, adding a single screening ultrasound to mammography in women at elevated risk detected an additional 4.2 cancers per 1000 (95% CI, 1.1–7.2) but, in the investigators’ own words, substantially increased the number of false positives, with a low positive predictive value for ultrasound-prompted biopsy [29]. Across three annual rounds, ultrasound added roughly 4.3 cancers per 1000 per year, yet only about 16% of the biopsies prompted by the combined mammography-plus-ultrasound examination proved malignant (PPV3 0.16, 95% CI 0.12–0.21) [30]. A new, deliberately sensitive non-mass lesion category is likely to behave similarly unless biopsy thresholds are calibrated against yield. Part of this burden is structural to BI-RADS itself: Category 3 and Category 4A trigger short-interval follow-up or biopsy despite low event rates; therefore, refining the downgrade rules for Category 3 and tightening biopsy criteria for 4A is one of the more direct ways to curb over-detection [28]—a point that connects to the inclusion of Category 3 in the v2025 audit. Overdiagnosis compounds the concern: in biennial screening of women aged 50–74, an estimated 15.4% of screen-detected cancers are overdiagnosed (95% uncertainty interval, 9.4–26.5), about one third of it attributable to the detection of nonprogressive (indolent) cancer [31], and more sensitive contrast-based detection of non-mass enhancement and enhancing asymmetry risks adding to this fraction. The health-economic picture is correspondingly unsettled. A recent systematic review with economic modeling found that supplemental MRI detects the most additional cancers (about 15–22 per 1000) and that CEM is comparable on limited evidence, whereas tomosynthesis and ultrasound add only 2–3 per 1000; cost-effectiveness results were heterogeneous, with MRI favorable only under specific assumptions and several analyses judging MRI or abbreviated MRI as not cost-effective, while CEM may be the more economical option but rests on sparse data [32]. At the system level, over-detection already accounts for substantial expenditure—on the order of USD 4 billion annually, much of it driven by false positives [28]. These trade-offs are not an argument against v2025 but a reason to apply it deliberately, and the Manual supplies the means to do so; the Method of Detection field and the structured exam indications give institutions, for the first time, a modality-neutral way to monitor the false-positive, biopsy, and cost consequences of each supplemental test, rather than assuming that more sensitivity is always better.

### 10.3. Resource-Stratified Implementation: A Global Health Perspective

A framework that assumes access to contrast-enhanced mammography, high-field MRI, and automated whole-breast ultrasonography describes only part of the world’s practice. Five-year breast-cancer survival exceeds 90% in high-income countries but falls to 66% in India and 40% in South Africa, and the WHO Global Breast Cancer Initiative was established in 2021 with the goal of reducing breast-cancer mortality by 2.5% per year—about 2.5 million lives by 2040—built on three pillars with measurable targets: more than 60% of invasive cancers diagnosed at stage I or II, diagnostic imaging and tissue sampling completed within 60 days, and more than 80% of patients completing multimodality treatment [33]. Within this framework, ultrasound, with or without diagnostic mammography, is the accessible diagnostic backbone where mammography is limited, and training sonographers is the recommended response to gaps in imaging capacity [33]. Resource-stratified guidance offers a way to map v2025 onto these realities rather than applying it uniformly: established frameworks tier recommendations into basic, core, and enhanced resource levels, so that each setting adopts what its capacity allows while retaining a path toward higher-level care [34]. On this logic, the resource-neutral elements of v2025—the cross-modality harmonization of shape, margin, and distribution terms, the least-to-most-suspicious ordering, and the structured exam-indication system—can be implemented essentially anywhere at minimal cost; ultrasound-based descriptors, including the new non-mass lesion category and elastography, sit at an intermediate level; and CEM and multiparametric MRI belong to higher-resource tiers. Implementation also depends on more than equipment: patient navigation, the first technical derivative of the GBCI implementation framework, is an evidence-informed means of overcoming access barriers in lower-resource settings [35]. Framed this way, v2025 need not be an all-or-nothing standard; its greatest global value may lie in giving a small district hospital and a tertiary referral centre a common vocabulary for the modalities each can actually provide.

## 11. Conclusions

Three contributions stand out. Terminology has gained a common vocabulary across modalities, with historically confusing terms such as microlobulated, milk of calcium, developing asymmetry, and initial phase clarified. Shape, margin, and distribution terms across mammography, ultrasonography, MRI, and CEM are more closely aligned, supporting multidisciplinary communication. The rewritten Category 6 wording, the “uncoupled” principle, the structured exam-indication system, and MOD give clinical decision processes a more flexible, data-supported structure. In daily practice, the same imaging finding is now read within a more consistent cross-modality vocabulary, with broader management options and stronger audit support. For practice, the priorities are concrete: updating reporting templates to the new lexicon—CEM reports and the revised calcification terms first—and pairing the least reproducible descriptors, such as non-mass enhancement and the recombined CEM features, with structured training and calibration. Because the harmonized terminology and structured indications cost little to implement while CEM and multiparametric MRI remain higher-resource additions, the framework can be adopted in a resource-stratified way, with the Method of Detection field used from the outset to track the balance between added detection and the false-positive, biopsy, and cost burden of each supplemental test. These conclusions apply to v2025 as an updated reporting framework; whether the revisions measurably improve reproducibility and diagnostic performance is still an open question, and answering it will require the prospective, modality-specific evidence outlined above. Looking further ahead, artificial intelligence and radiomics offer the most credible route to the reproducibility the lexicon alone cannot guarantee, and an electronic, continuously revisable format should let the system keep pace; its full value, however, will depend as much on equitable implementation across diverse practice settings as on this evidence, so that a globally standardized lexicon translates into globally comparable care.

## Figures and Tables

**Table 1:** Chronology of BI-RADS^®^ Editions and Structural Comparison Between the 2013 Atlas and v2025 Manual.

A. Chronology of BI-RADS^®^ Editions			
Year	Version	Modalities Covered	Principal Innovation
1992	1st Edition	Mammography	First lexicon, reporting structure, and audit framework
1995	2nd Edition	Mammography	Lexicon revision
1998	3rd Edition	Mammography	Atlas of artist illustrations added
2003	4th Edition	Mammography + US + MRI	US and MRI lexicons added; Category 4 split into 4A/B/C
2013	5th Edition/Atlas	Mammography + US + MRI	Percentage-based density removed; cross-modality terminology alignment; some vague terms removed from the lexicon
2025	v2025/Manual	Mammography + US + MRI + CEM	“Atlas” → “Manual”; edition number → year-based versioning; CEM as full section; “microlobulated” margin descriptor removed; DBT/AWBUS; structured indications
**B. Structural Comparison Between the 2013 BI-RADS^®^ Atlas and the ACR BI-RADS^®^ v2025 Manual**			
**Feature**		**2013 BI-RADS^®^ Atlas (5th Edition)**	**ACR BI-RADS^®^ v2025 Manual**
**Document scope and versioning**		**Visual atlas, edition-numbered**	**Definitions, guidance, and reporting framework; year-based versioning**
**Sections**		4 (Mammography, US, MRI, Auditing)	5 (Mammography, US, MRI, CEM, AOM)
**CEM**		Supplement only	Full section (5th section)
**Mammography margin descriptors**		Circumscribed, Obscured, Microlobulated, Indistinct, Spiculated	Microlobulated removed; Circumscribed, Obscured, Indistinct, Spiculated
**New imaging technologies**		Not included	DBT, synthetic mammography, AWBUS examples
**Lexicon ordering principle**		Not specified	Least suspicious to most suspicious
**Reporting organization**		Standardized across modalities	Harmonized across modalities, plus structured exam indication
**Breast density notification**		a–d categories only	a–d plus 2024 FDA mandatory notification plus 2025 FDA singular-wording standard

**Table 2:** Mammography Lexicon: Comparison of the 2013 BI-RADS^®^ 5th Edition with ACR BI-RADS^®^ v2025.

Section A—Breast Density				
Category	2003 Definition (% Values)	2013 Definition	v2025 Additions	Rationale
**a**	<25% fibroglandular tissue	Almost entirely fatty	Preserved	Volume percentage inconsistent with observer agreement
**b**	25–50%	Scattered areas of fibroglandular density	Preserved	Masking potential as the defining principle
**c**	50–75%	Heterogeneously dense; may obscure small masses	Preserved	
**d**	>75%	Extremely dense; lowers mammographic sensitivity	2024 FDA mandatory notification plus 2025 alternative standard; OR 1.6 cancer risk; CDR with DBT 4.5 → 5.8 per 1000	
**Section B—Mass Descriptors**				
**Descriptor**	**Type**	**2013 Status**	**v2025 Status**	**Rationale**
**Oval**	Shape	Present	Preserved	
**Lobulated**	Shape	Absent (removed in 4th edition)	REINTRODUCED; indented or wavy contour, slightly more suspicious than oval	More precise communication; terminology alignment across the three modalities
**Round**	Shape	Present	Preserved	
**Irregular**	Shape	Present	Preserved	
**Circumscribed**	Margin	Present	Preserved	
**Obscured**	Margin	Present	Preserved	
**Microlobulated**	Margin	Present, suspicious finding	REMOVED	Terminological confusion with “lobulated” shape
**Indistinct**	Margin	Present	Preserved	
**Spiculated**	Margin	Present	Preserved	
**High/equal/low/fat-containing**	Density	Present	Preserved	
**Mass definition on a single projection**	Definition criterion	Not valid; two projections required	Valid on a single DBT projection	Reduced fibroglandular superposition with DBT
**Section C—Calcification Terminology (with Malignancy Risk)**				
**Descriptor**	**Category**	**2013 Status**	**v2025 Status**	**Malignancy Risk (PPV)**
**Skin/Vascular/Large rod-like/Suture**	**Typically benign**	**Present**	**Preserved**	**~0% [13,14]**
**Coarse/popcorn-like**	**Typically benign**	**Present**	**Preserved (dystrophic placed within this heading)**	**~0% [13,14]**
**Round**	**Typically benign**	**Present, with “punctate” as a separate subgroup**	**Present; punctate folded into “round”**	**9% (punctate)**
**Milk of calcium**	**Typically benign**	**Present**	**Present; designated “layering”**	**~0% [13,14]**
**Rim**	**Typically benign**	**Present (eggshell and lucent-centered consolidated)**	**Preserved**	**~0% [13,14]**
**Dystrophic**	**Typically benign**	**Separate descriptor**	**Within “coarse” category**	**~0% [13,14]**
**Amorphous**	**Suspicious (4B)**	**Present**	**Preserved**	**13–26% [13,14]**
**Coarse heterogeneous**	**Suspicious (4B)**	**Present**	**Preserved**	**7–20% [13,14]**
**Fine pleomorphic**	**Suspicious (4B)**	**Present**	**Preserved**	**28–29% [13,14]**
**Fine linear/linear-branching**	**Suspicious (4C)**	**Present**	**Preserved**	**53–70% (up to 81% for linear morphology) [13,14]**
**Clustered distribution upper limit**	**Distribution**	**1 cm**	**2 cm**	**Clustered 22–36%; segmental 74%, linear 68% [14]**
**Section D—Asymmetry Categories**				
**Asymmetry Type**	**Definition**	**2013 Status**	**v2025 Status**	**PPV/Clinical Relevance**
**Asymmetry**	One projection only; usually superposition	Present	Preserved	Generally normal tissue overlap
**Focal asymmetry**	Two projections; no convex contour or central density; interspersed fat	Present	Preserved	Screening PPV 7.4–12.8%; diagnostic 19.7–26.7%
**Global asymmetry**	≥1 quadrant; no matching structure	Present	Preserved	Usually a normal variant; assess with clinical findings
**Developing asymmetry**	New, growing, or more conspicuous than on prior study	Present, independent descriptor	REMOVED AS DESCRIPTOR; clinical importance preserved; new or increasing focal asymmetry remains an indication for biopsy	Biopsy PPV2 up to 42.9%; cancer detection rate 1.6 per 1000 in screening

Abbreviations and notes: PPV, positive predictive value. Positive predictive values come from a full-field digital mammography series of 146 lesions with an overall PPV of 28.8% [14], an earlier series of 115 lesions with an overall PPV of 21.7% [13], and a categorical analysis of 492 lesions. Fine linear and linear-branching morphology carried a significantly higher malignancy risk than the other suspicious descriptors (*p* < 0.001 [14]; *p* = 0.005 [13]). Ranges reflect the spans reported across these sources.

**Table 3:** Ultrasound Lexicon: Comparison of the 2013 BI-RADS^®^ 5th Edition with ACR BI-RADS^®^ v2025.

Section A—Mass Descriptors					
Parameter	Descriptor	2013 Status	v2025 Status	Rationale	
Shape	Oval	Present	Preserved		
	Lobulated	Absent	REINTRODUCED; indented or wavy contour, slightly more suspicious than oval	Terminological coherence across three modalities; more precise communication	
	Round	Present	Preserved		
	Irregular	Present	Preserved		
Orientation	Parallel to skin	Present	Preserved		
	Not parallel to skin	Present (formerly “taller-than-wide”)	Preserved		
Margin	Circumscribed	Present	Preserved		
	Indistinct	Present	Preserved		
	Angular	Present	Preserved		
	Microlobulated	Present	Preserved		
	Spiculated	Present	Preserved		
Echo pattern	Anechoic	Present	Preserved		
	Hypoechoic	Present	Preserved		
	Isoechoic	Present	Preserved		
	Hyperechoic	Present	Preserved		
	Complex cystic and solid	Present	Preserved		
	Heterogeneous	Present	Preserved		
	Mixed solid and cystic	Absent	REINSTATED	Emphasizing the importance of the solid component; conceptual distinction from “complex cystic and solid”	
Posterior features	None/Enhancement/Shadowing/Combined	Present	Preserved		
Section B—Non-Mass Lesion (New v2025 Category)					
Parameter	**Options**			**2013 Counterpart**	**v2025 Status**
Distribution	Focal			Absent	New
	Linear			Absent	New
	Segmental			Absent	New
	Regional			Absent	New
	Diffuse			Absent	New
Echo pattern	Anechoic			Absent	New
	Hypoechoic			Absent	New
	Heterogeneous			Absent	New
	Hyperechoic			Absent	New
Clinical relevance	Mammographic focal asymmetry/MRI NME counterpart; infiltrative cancers; biopsy guidance for DBT/MBI/CEM			Absent	New addition
Section C—Ultrasound Associated Features (with Elastography Performance)					
Feature	**2013 Status**	**v2025 Status**	**Diagnostic Performance**		
Architectural distortion	Present	Preserved			
Ductal changes	Present	Preserved			
Skin thickening/retraction	Present	Preserved			
Edema	Present	Preserved			
Vascularity (absent/internal/rim)	Present	Preserved			
Elastography (soft/intermediate/hard)	Present	Preserved	Compression elastography with B-mode: sensitivity 99.0% (99/100), specificity 91.5% (119/130) [17]. Shear-wave elastography AUC 0.928 (95% CI 0.886–0.970) versus 0.851 (95% CI 0.791–0.911) for B-mode alone [18].		
Echogenic rind	Absent	Present	Echogenic halo reflecting peritumoral tissue reaction; observed in some invasive cancers		
Section D—Lymph Node Assessment					
Parameter	**2013 Status**		**v2025 Status**		
Normal intramammary LN criteria	Fatty hilum, oval/kidney shape, generally <1 cm		Preserved plus cortex ≤ 3 mm criterion specified		
Axillary LN	Under “special cases”		Expanded		
Suspicious findings for tumor involvement	Limited information		Spectrum of cortical thickening, eccentric cortex, hilar effacement, and rounding detailed		
Regional nodal basins	Axillary and intramammary only		Axillary Levels I–II–III plus Internal Mammary plus Supraclavicular plus Infraclavicular		
TNM nodal staging correlation	Absent		Role of each basin in TNM defined		

Abbreviations and notes: AUC, area under the receiver operating characteristic curve; CI, confidence interval. Elastography performance reflects a community-based series of 230 elastograms [17] and a prospective comparison of 150 lesions in which shear-wave elastography was more reproducible and operator-independent than strain elastography [18]. On shear-wave elastography, malignant lesions were stiffer than benign lesions (150.0 ± 52.3 kPa versus 47.3 ± 44.3 kPa; *p* < 0.0001) [18].

**Table 4:** MRI Lexicon: Comparison of the 2013 BI-RADS^®^ 5th Edition with ACR BI-RADS^®^ v2025.

Section A—Mass Descriptors				
Parameter	Descriptor	2013 Status	v2025 Status	Rationale
Shape	Oval	Present	Preserved	
	Lobulated	Absent (removed in 4th edition)	REINTRODUCED; indented or wavy contour, slightly more suspicious than oval	Terminological coherence across three modalities
	Round	Present	Preserved	
	Irregular	Present	Preserved	
Margin	Circumscribed	Present (renamed from “smooth” in 2013)	Preserved	
	Not circumscribed: irregular	Present	Preserved	
	Not circumscribed: spiculated	Present	Preserved	
Internal enhancement	Homogeneous	Present	Preserved	
	Heterogeneous	Present	Preserved	
	Rim enhancement	Present	Preserved	
	Dark internal septations	Present	Preserved	
T2 signal intensity	Hyperintense/Not hyperintense	Absent	NEWLY ADDED	Contribution to lesion characterization
Section B—Kinetic Curve Terminology				
Parameter	**2013 Status**	**v2025 Status**	**Rationale**	
Early phase name	“Initial phase”	“Early phase”	Multiple early time points obtainable with fast MRI protocols; ambiguity of “initial”	
Early phase: slow	Slow	Preserved		
Early phase: medium	Medium	Preserved		
Early phase: rapid	Rapid	Preserved		
Late phase: persistent	Persistent	Preserved		
Late phase: plateau	Plateau	Preserved		
Late phase: washout	Washout	Preserved		
Abbreviated MRI protocol	Absent	Introduced	Practicality in screening and clinical use	
Section C—Non-Mass Enhancement (NME) Terminology				
Subcategory	**Descriptor**	**2013 Status**	**v2025 Status**	**Basis**
Distribution	Diffuse	Present	Preserved	
	Regional	Present	Preserved	
	Focal	Present	Preserved	
	Linear	Present	Preserved	
	Segmental	Present	Preserved	PPV 62.5%, with moderate inter-observer agreement (κ 0.67–0.69) under the fifth-edition lexicon [4]
	Ductal	Dropped in 2013	Absent	Low usage
Internal enhancement	Homogeneous	Present	Preserved	
	Heterogeneous	Present	Preserved	
	Clumped	Present	Preserved	
	Clustered ring	Added in 2013	Preserved	Suspicious periductal stromal enhancement; PPV 53.85%, moderate inter-observer agreement (κ 0.67–0.69), fifth-edition lexicon [4]
	Reticular/dendritic	Dropped in 2013	Absent	Low usage
	Stippled/punctate	Dropped in 2013	Absent	Conceptual overlap with BPE
Symmetry descriptors	Symmetric/asymmetric	Within NME	Moved to the BPE category	Simplification of the NME definition
Section D—Implant Terminology				
Descriptor			**2013 Status**	**v2025 Status**
Retroglandular location			Added in 2013	Preserved
Retropectoral location			Added in 2013	Preserved
Focal bulge			Added in 2013	Preserved
Radial folds			Added in 2013	Preserved
Subcapsular line			Added in 2013	Preserved
Keyhole sign			Added in 2013	Preserved
Linguine sign			Added in 2013	Preserved
Extracapsular silicone: lymph node			Added in 2013	Preserved
Extracapsular silicone: breast			Added in 2013	Preserved
Water droplets			Added in 2013	Preserved
Peri-implant fluid			Added in 2013	Preserved
Material/lumen type classification			Added in 2013	Preserved
Abbreviated implant MRI protocol guidance			Absent	NEWLY ADDED

Abbreviations and notes: BPE, background parenchymal enhancement; κ, Cohen kappa coefficient; NME, non-mass enhancement; PPV, positive predictive value. When clustered ring internal enhancement and segmental distribution occur together, the reported positive predictive value reaches 66.67%; these values come from prospective data using the fifth-edition lexicon [4].

**Table 5:** CEM Lexicon and Comparison of BI-RADS Assessment Categories: 2013 vs. v2025.

Section A—CEM Lexicon: Comparison of Mammography, MRI, and CEM Terminology				
Finding Category	Parameter	Mammography (2013)	MRI (2013/v2025)	CEM v2025
**Background Enhancement (BPE)**	Level	Not applicable	Minimal/Mild/Moderate/Marked	Minimal/Mild/Moderate/Marked
	Symmetry	Not applicable	Symmetric/Asymmetric	Symmetric/Asymmetric
**Mass: Shape**	Oval	✓	✓	✓
	Lobulated	Absent in 2013, added in v2025	Absent in 2013, added in v2025	Added in v2025
	Round	✓	✓	✓
	Irregular	✓	✓	✓
**Mass: Margin**	Circumscribed	✓	✓	✓
	Not circumscribed: indistinct	✓	✓	✓ (“irregular” updated to “indistinct”)
	Not circumscribed: spiculated	✓	✓	✓
**Mass: Internal enhancement**	Homogeneous	Not applicable	✓	✓
	Heterogeneous	Not applicable	✓	✓
	Rim enhancement	Not applicable	✓	✓
**NME: Distribution**	Diffuse	Not applicable	✓	✓
	Regional	Not applicable	✓	✓
	Focal	Not applicable	✓	✓
	Linear	Not applicable	✓	✓
	Segmental	Not applicable	✓	✓
	Multiple regions	Not applicable	Removed in v2025	Absent (MRI alignment)
**Special findings**	Enhancing asymmetry	Not applicable	Not applicable	✓ (CEM-specific)
**Section B—BI-RADS Assessment Categories: 2013 vs. v2025**				
**Category**	**2013 Definition**	**v2025 Update**	**Probability of Malignancy**	**Management Recommendation**
**0**	Incomplete; additional imaging or prior films needed	Preserved		Recall/comparison
**1**	Negative; no significant finding	Preserved	~0%	Routine screening
**2**	Benign finding	Preserved	~0%	Routine screening
**3**	Probably benign	Preserved; not applicable in screening mammography	>0% to ≤2%	6-month short-interval follow-up
**4A**	Suspicious; low suspicion	Preserved	>2% to ≤10%	Tissue diagnosis
**4B**	Suspicious; moderate suspicion	Preserved	>10% to ≤50%	Tissue diagnosis
**4C**	Suspicious; high suspicion	Preserved	>50% to <95%	Tissue diagnosis
**5**	Highly suggestive of malignancy	Preserved	≥95%	Tissue diagnosis
**6**	Biopsy-proven malignancy	UPDATED: surgery is not the only definitive local treatment option		Clinical follow-up; definitive local therapy (usually surgical, but not the only option)

Together, the Category 6 revision, the “uncoupled” principle, and the linkage to structured indications turn assessment categories from a malignancy-probability scheme into a structure that also carries clinical context, multidisciplinary decisions, and cross-modality outcome tracking.

**Table 6:** Quantitative Performance and Reproducibility Data for Key v2025 Lexicon Elements.

Lexicon Element	Modality	Performance Metric	Value	Source	Source Year
**Extremely dense breast (Category D)**	Mammography	Odds ratio for cancer	1.6	[5]	2025
**Dense breast, DBT**	Mammography	Cancer detection rate	4.5 to 5.8 per 1000	[5]	2025
**Spiculated margin**	Mammography	PPV	81% (56/69)	[20]	1998
**Indistinct margin**	Mammography	PPV	44% (29/66)	[20]	1998
**Irregular shape**	Mammography	PPV	73% (66/90)	[20]	1998
**Fine linear/linear-branching calcifications**	Mammography	PPV	53–70% (up to 81% for linear)	[13,14,20]	1998–2010
**Fine pleomorphic calcifications**	Mammography	PPV	28–29%	[13,14]	2007–2010
**Amorphous calcifications**	Mammography	PPV	13–26%	[13,14,20]	1998–2010
**Coarse heterogeneous calcifications**	Mammography	PPV	7–20%	[13,14]	2007–2010
**Segmental/linear calcification distribution**	Mammography	PPV	68–74%	[20]	1998
**Focal asymmetry**	Mammography	PPV (screening/diagnostic)	7.4–12.8%/19.7–26.7%	[2,5,15]	2007–2025
**Enlarging/more conspicuous focal asymmetry**	Mammography	Biopsy PPV_2_; cancer detection rate	42.9%; 1.6 per 1000	[15]	2007
**Compression elastography with B-mode**	US	Sensitivity/specificity	99.0%/91.5%	[17]	2013
**Shear-wave elastography**	US	AUC (vs. B-mode 0.851)	0.928 (95% CI 0.886–0.970)	[18]	2013
**BPE moderate/marked vs. minimal/mild**	MRI	DCE-MRI sensitivity	88% vs. 99% (Δ 11%; 95% CI 3.7–19; *p* = 0.0058)	[19]	2016
**Clustered ring internal enhancement (NME)**	MRI	PPV; inter-observer agreement (κ)	53.85%; 0.67–0.69	[4]	2020
**Segmental distribution (NME)**	MRI	PPV; inter-observer agreement (κ)	62.5%; 0.67–0.69	[4]	2020
**Clustered ring with segmental distribution (NME)**	MRI	Combined PPV	66.67%	[4]	2020
**Breast density and lesion type, LE images**	CEM	Inter-reader agreement (Fleiss κ)	density 0.569 (moderate); lesion type 0.654 (substantial); microcalcifications 0.820 (almost perfect)	[9]	2025
**Type of enhancement, RC images**	CEM	Inter-reader agreement (Fleiss κ)	overall 0.664 (substantial); mass 0.733; non-mass 0.541; enhancing asymmetry 0.320	[9]	2025
**Mass enhancement descriptors, RC images**	CEM	Inter-reader agreement (Fleiss κ)	shape 0.523; margin 0.566; internal pattern 0.618	[9]	2025
**Non-mass enhancement and enhancing asymmetry, RC images**	CEM	Inter-reader agreement (Fleiss κ)	NME distribution 0.387 (fair); NME internal pattern 0.417 (moderate); enhancing asymmetry 0.247 (fair)	[9]	2025
**Final BI-RADS assessment**	CEM	Inter-reader agreement (Fleiss κ); reader sensitivity	LE 0.421, CEM 0.364; sensitivity 88–91% with CEM	[9]	2025

**Abbreviations and notes:** AUC, area under the receiver operating characteristic curve; BPE, background parenchymal enhancement; CEM, contrast-enhanced mammography; CI, confidence interval; DBT, digital breast tomosynthesis; DCE, dynamic contrast-enhanced; κ, kappa coefficient; LE, low-energy; MRI, magnetic resonance imaging; NME, non-mass enhancement; PPV, positive predictive value; PPV_2_, positive predictive value for biopsy recommendation; RC, recombined; US, ultrasonography. Kappa values for contrast-enhanced mammography are Fleiss coefficients from three readers evaluating 462 lesions, interpreted on the Landis and Koch scale (0.21–0.40 fair, 0.41–0.60 moderate, 0.61–0.80 substantial, 0.81–0.99 almost perfect) [9]. Agreement was lowest for non-mass enhancement and enhancing asymmetry, although this did not reduce diagnostic performance. Other ranges reflect the spans published across the cited sources. The Source year column gives the publication year of each primary source. Most values predate v2025 and come from the fifth-edition lexicon or the first CEM lexicon, not from v2025 itself. The 1998 source is retained because no later study has reproduced these descriptor-level PPVs [20].

## Data Availability

Data is contained within the article or Supplementary Material.

## Supplementary Materials

The following supporting information can be downloaded at https://www.mdpi.com/article/10.3390/diagnostics16132135/s1. Table S1: Literature Search Strategy; Table S2: Completed SANRA Checklist.

## Abbreviations

The following abbreviations are used in this manuscript:

ACR	American College of Radiology
AIR	Abnormal Interpretation Rate
AOM	Auditing and Outcomes Monitoring
AWBUS	Automated Whole-Breast Ultrasonography
BI-RADS	Breast Imaging Reporting and Data System
BPE	Background Parenchymal Enhancement
CC	Cranio-Caudal
CDR	Cancer Detection Rate
CEM	Contrast-Enhanced Mammography
DBT	Digital Breast Tomosynthesis
DCE-MRI	Dynamic Contrast-Enhanced Magnetic Resonance Imaging
EUSOBI	European Society of Breast Imaging
FAQ	Frequently Asked Questions
FDA	Food and Drug Administration
LE	Low-Energy
MBI	Molecular Breast Imaging
MLO	Medio-Lateral Oblique
MOD	Method of Detection
MQSA	Mammography Quality Standards Act
MRI	Magnetic Resonance Imaging
NME	Non-Mass Enhancement
PPV	Positive Predictive Value
PRISMA	Preferred Reporting Items for Systematic Reviews and Meta-Analyses
RC	Recombined
SM	Synthetic Mammography
TNM	Tumor, Node, Metastasis
US	Ultrasonography
