# Supplementary material for: How Does ACR BI-RADS® v2025 Change the Radiologist’s Approach? A Practical Guide Across Mammography, Ultrasound, and MRI: A Narrative Review
Source: Diagnostics (Basel). 2026 Jul 7;16(13):2135. doi: 10.3390/diagnostics16132135 (PMC13361901; doi:10.3390/diagnostics16132135)
Supplement: Supplementary file 1 [file diagnostics-16-02135-s001.zip › diagnostics-4394100-supplementary.pdf]

Supplementary Materials

How Does ACR BI-RADS® v2025 Change the Radiologist's Approach? A Practical Guide Across Mammography, Ultrasound, and MRI: A Narrative Review

Table S1. Literature Search Strategy

A reproducible literature search was carried out in PubMed (MEDLINE) and combined with hand-searching (backward citation chasing) of the reference lists of included articles. The two source documents — the 2013 ACR BI-RADS® Atlas (5th edition) and the ACR BI-RADS® v2025 Manual — were obtained directly from the American College of Radiology and were not part of the database search. Because this is a narrative synthesis, no PRISMA-style record-count flow was applied; reproducibility rests on the exact query strings, fields, and dates reported below.

Parameter	Specification
Database / platform	PubMed (MEDLINE), via pubmed.ncbi.nlm.nih.gov
Supplementary searching	Hand-search (backward citation chasing) of the reference lists of included articles
Source documents	2013 ACR BI-RADS® Atlas (5th edition); ACR BI-RADS® v2025 Manual (obtained directly from the ACR)
Publication window	1998–2025
Date search last run	December 2025
Language	English only
Eligible article types	Original research, systematic reviews, meta-analyses, and society / consensus guidelines
Excluded	Conference abstracts, editorials, and isolated case reports (unless they directly informed lexicon use)
Reporting standard	Narrative synthesis; PRISMA 2020 not applicable

Search strings (entered as separate PubMed search-history lines and then combined):

#1 Concept: BI-RADS / breast imaging lexicon

```
"BI-RADS"[Title/Abstract] OR BIRADS[Title/Abstract] OR "Breast Imaging Reporting and Data System"[Title/Abstract] OR "breast lexicon"[Title/Abstract] OR "breast imaging lexicon"[Title/Abstract]
```

#2 Concept: breast imaging modalities

```
"Mammography"[Mesh] OR mammography[Title/Abstract] OR "digital breast tomosynthesis"[Title/Abstract] OR tomosynthesis[Title/Abstract] OR "synthetic mammography"[Title/Abstract] OR "Ultrasonography, Mammary"[Mesh] OR "breast ultrasound"[Title/Abstract] OR "breast ultrasonography"[Title/Abstract] OR "automated breast ultrasound"[Title/Abstract] OR "breast MRI"[Title/Abstract] OR "breast magnetic resonance imaging"[Title/Abstract] OR "contrast-enhanced mammography"[Title/Abstract] OR "contrast-enhanced spectral mammography"[Title/Abstract]
```

#3 Concept: lexicon descriptors and reader performance

```
calcification*[Title/Abstract] OR microcalcification*[Title/Abstract] OR "mass margin"[Title/Abstract] OR "non-mass enhancement"[Title/Abstract] OR "background parenchymal enhancement"[Title/Abstract] OR "breast density"[Title/Abstract] OR elastography[Title/Abstract] OR asymmetry[Title/Abstract] OR "positive predictive value"[Title/Abstract] OR interobserver[Title/Abstract] OR "inter-observer"[Title/Abstract] OR reproducibility[Title/Abstract] OR "reader agreement"[Title/Abstract] OR kappa[Title/Abstract]
```

#4 Combined core

```
#1 AND #2
```

#5 Focused (core + performance)

```
#1 AND #2 AND #3
```

#6 Concept: regulation, guidelines, and screening

```
(#1) AND ("United States Food and Drug Administration"[Mesh] OR FDA[Title/Abstract] OR "breast
density notification"[Title/Abstract] OR EUSOBI[Title/Abstract] OR "structured
reporting"[Title/Abstract] OR guideline*[Title/Abstract] OR recommendation*[Title/Abstract] OR
screening[Title/Abstract])
```

#7 Final set (eligibility filter applied)

```
(#5 OR #6) AND English[Language]
```

Screening: titles and abstracts were screened for relevance to multimodality breast-imaging terminology (the 2013 fifth edition and the v2025 changes). Full texts of potentially relevant records were assessed against the eligibility criteria above, and the reference lists of included articles were hand-searched to capture foundational descriptor- and predictive-value studies not returned by the primary search. Records outside the 1998–2025 window were excluded at screening.

Mapping of concept blocks to the references included in the review (illustrating retrieval coverage):

Topic block in the review	Included references retrieved
BI-RADS history, structure, and editions	Burnside 2009 [1]; Zonderland 2014 [2]; Spak 2017 [3]; D'Orsi BI-RADS Atlas, 5th ed. 2013 [10]; ACR BI-RADS v2025 Manual [5]
Mammographic descriptors and feature-level PPV	Liberman 1998 [20]; Burnside 2007 [13]; Bent 2010 [14]; Leung & Sickles 2007 [15]; Chang 2010 [16]
Breast density and patient notification	Morrish 2015 [11]; FDA MQSA final rule 2024 [12]
Ultrasound and elastography	Destounis 2013 [17]; Chang 2013 [18]
MRI: NME, BPE, and interobserver agreement	Lunkiewicz 2020 [4]; Telegrafo 2016 [19]
Contrast-enhanced mammography	Covington 2024 [6]; Zarcaro 2025 [9]
Structured reporting, screening, and society guidance	ESR 2023 [7]; Marcon / EUSOBI 2024 [8]

Table S2. Completed SANRA Checklist

The manuscript was self-assessed against the SANRA (Scale for the Assessment of Narrative Review Articles) instrument. Each of the six items is scored from 0 (low) to 2 (high), for a maximum of 12.

#	SANRA criterion	Score (0–2)	How it is addressed in the revised manuscript
**1**	Justification of the article's importance for the readership	**2**	The Introduction states that v2025 is the first BI-RADS revision in twelve years, that it now spans four modalities including the newly independent CEM section, and that most existing literature is single-modality or structural; the value of the review for radiologists adopting v2025 is made explicit.

#	SANRA criterion	Score (0–2)	How it is addressed in the revised manuscript
**2**	Statement of concrete aims or formulation of questions	**2**	The aim is stated at the end of the Introduction (with Table 1): to compare the 2013 fifth edition with v2025 across mammography, ultrasonography, MRI, and CEM, and to relate each main change to the available diagnostic-performance and reader-agreement data, using the Manual as a reference framework rather than the sole source.
**3**	Description of the literature search	**2**	The Methods report the database (PubMed), the search terms, the publication window (1998–2025), the last-run year, the hand-searching of reference lists, and the inclusion / exclusion criteria; the full, reproducible search strings are provided in Table S1.
**4**	Referencing	**2**	Claims are supported by primary studies, society guidelines, and the two source manuals. Where performance figures are cited, the study design, sample size, and main limitations are noted (Methods; Tables 2–6).
**5**	Scientific reasoning (incorporation of appropriate evidence)	**2**	Each change is read against PPV and reader-agreement (κ) data rather than only catalogued. The review states explicitly that most supporting data predate v2025, that reproducibility is lower for newer descriptors (e.g., non-mass enhancement, enhancing asymmetry), and identifies the specific descriptors that still lack validation (Discussion; Table 6).
**6**	Appropriate presentation of data	**2**	Quantitative results are reported with denominators, confidence intervals, p-values, and κ interpretation where available, and are synthesized — with source years — in a dedicated table (Tables 2–6, especially Table 6; Figure 2).
	**Total score**	**12 / 12**	Maximum attainable score = 12.

SANRA reference: Baethge C, Goldbeck-Wood S, Mertens S. SANRA — a scale for the quality assessment of narrative review articles. Research Integrity and Peer Review. 2019;4:5.
